# Estimating the non-linear effects of urban built environment at residence and workplace on carbon dioxide emissions from commuting

**DOI:** 10.3389/fpubh.2022.1077560

**Published:** 2022-11-29

**Authors:** Qingchun Liu, Peixiong Zhao, Yingying Zhang, Zhihui Zhang, Jun Yang

**Affiliations:** ^1^School of Economics, Shandong University of Finance and Economics, Jinan, China; ^2^Policy Research Office, Shandong Academy of Social Sciences, Jinan, China; ^3^UniSA Business, University of South Australia, Adelaide, SA, Australia; ^4^School of Humanities and Law, Northeastern University, Shenyang, China; ^5^Jangho Architecture College, Northeastern University, Shenyang, China; ^6^Human Settlements Research Center, Liaoning Normal University, Dalian, China

**Keywords:** built environment, gradient boosting decision model, CO_2_ emissions from commuting, non-linear relationship, threshold effect

## Abstract

Understanding the relationship between CO_2_ emissions from commuting (CEC) and the built environment is crucial for sustainable transportation and land-use policymaking during the process of constructing a low carbon city. Previous studies usually assume that the relationship is linear, which may lead to inaccurate CEC prediction and ineffective policy. Using daily travel survey data of residents in the central city of Jinan, this study adopted a gradient boosting decision tree model to explore the threshold effect and the non-linear relationship between built environments and CEC. Our findings suggest that 40% of CEC is related to the workplace environment, which is higher than the residential environment and other socioeconomic variables. The five most important variables are road density within 1 km radius of the workplace (13.493%), distance to the center at workplace and residence (10.908%, 10.530%), population density at workplace (9.097%) and distance to bus stop from the residence (8.399%). Distance to city center plays the most important role and its non-linear relationship reflects the influence of the urban spatial structure of Jinan on CEC. Furthermore, the thresholds and non-linear relationships provide planning guidelines to support urban planning development policies for low carbon city.

## Introduction

As an important source of energy consumption, the transportation sector accounts for a substantial share of greenhouse gas (GHG) emissions (1, 2). Due to rapid urbanization and motorization, developing countries have higher transport-related energy consumption and GHG emissions (3). However, rapid urbanization and motorization brought along consequences such as heavy usage of vehicles, traffic congestion, road accidents, and air pollution in many major cities in China, which has a negative impact on the health of residents (4). Since commuting is an essential daily activity for urban residents, which accounts for approximately 50%~60% of their total trips (5), it is necessary to develop methods to control CEC effectively.

The urban built environment is the spatial reflection of land-use policies, and the source of urban traffic demand. It also has a locked effect on travel behaviors (the commuting mode, commuting distance, and travel times), implying that the urban built environment could also affect CEC. Urban geography and urban planning scholars have attempted to create green transportation-oriented urban built environments and minimize automobile dependence to reduce CEC by optimizing urban spatial organization. Western countries which have adopted transportation and land-use policies including new urbanism, smart growth, and transit-oriented development (TOD) programs to alleviate the problem of urban sprawl have achieved positive results. However, some research found that these policies were weakened for large cities in China due to the urban spatial structural transformation and the complexity of institution reform (6). While existing literature attaches great importance to the effect of the built environment at residence, the effect of the built environment at workplace is often neglected. However, the built environment characteristics at workplace may be associated with the commute behaviors (7) and hence changing the daily CEC. Workers are less likely to choose their jobs based on the built environment characteristics at workplaces, representing the independent effects of the built environment instead of self-selection effects. However, the effects of the built environment characteristics at workplaces on CEC rarely have been explicitly examined. If the built environment at workplace has a significant effect on CEC, physical improvements in residential areas are insufficient to achieve the goal of carbon reduction. Moreover, some studies using the machine learning methods reveal that there is non-linear relationship between built environment and travel behavior (8–11). This would help decision makers understand the threshold of the built environment variables, and improve the outcomes of the urban planning process. Therefore, understanding CEC and its relationship to the built environment at residence and workplace is crucial and necessary for sustainable effective transportation and land-use policymaking.

Using the daily travel survey data of residents of the central city of Jinan in China, this study contributes to the existing literature from the following aspects: (1) Gradient boosting decision tree (GBDT) model is applied to estimate the non-linear effects of the built environment on CEC at both residential and workplace locations, this would help to examine the threshold of built environment variables, and better understand the CEC problem, (2) this study analyzes the importance of built environment on CEC, (3) our results provide the empirical evidence toward the development of large cities in developing countries.

The remainder of the paper is structured as follows. Section 2 provides a brief review of the impact of built environments on CEC. Section 3 introduces the research design, including regional characteristics, data sources, and research methods. Section 4 presents the results. Section 5 discusses research limitations and possible future research directions. Section 6 concludes the paper.

## Literature

For over two decades, CEC has received significant attention. Urban built environments and socioeconomic factors have been considered to have significant impacts on CEC in previous studies. The urban built environment includes many factors, which referred to as 6D, i.e., density, diversity, design, destination accessibility, distance to bus stop, and demand management (9, 12). Above six factors are closely related to commuting behaviors. Based on aggregate (country/city) or disaggregate (individual/household) data, the direct and indirect impacts of different built environment variables on commuting behaviors and related CO_2_ emissions are already well explored, especially for the built environment variables at the residence (13–16). Using different methodological approaches and geographies, many research showed that a community has great potential to reduce automobile use and CEC if its built environment characteristics include a high population density, good public transport accessibility, and a high land-use diversity, although the significance and magnitude of the effects of these built environment factors can vary substantially (12, 16).

Many empirical studies were conducted to examine the influence of the built environment on CEC in residential areas. Based on data from the 2006 Austin Household Travel Survey, Choi and Zhang (17) found that a 1% increase in density was found to reduce household vehicle emissions by 0.1%. However, this relationship is not consistent due to the different geographical contexts. Other studies have found that when the residential population density reaches a certain level of threshold, improving the density will not necessarily reduce CEC. Using the 2006 Puget Sound Household Travel Survey data, Hong (18) found that people living in denser neighborhoods tend to generate fewer CEC. However, this effect becomes insignificant as population density reaches a certain level. Most studies confirmed that the mixed degree of land use is negatively correlated with CEC, and improving the degree of land use mixing in urban suburbs is more helpful for reducing CEC than improving population density (19, 20). Distance to bus stop access is positively related with CEC (21). However, Chai et al. (19) found that households located far from the city center but with greater transit accessibility in Beijing increased their dependence on public transportation, thereby reducing the probability of commuting by private car. Similarly, improving street connectivity in residential areas will reduce CEC (22, 23).

However, as behavioral research deepens our understanding of people's daily behaviors, several studies started to focus on the effect of the built environment in non-residential locations. Researchers believe that people's commuting will be jointly influenced by the built environment of their residences and workplaces. The built environment at the workplace appears to have an important impact on travel behavior, but it is overlooked in the literature. Considering that only the geographical environment of residential areas may lead to misunderstanding of the results, overestimating the background impact of neighborhood areas and underestimating the impacts of other places (24). Huang et al. (25) represented the overall geographical environment in a more accurate way. Furthermore, examining the influence of built environment variables at workplace will provide a reference for the design and redevelopment of employment centers. Otherwise, urban planners may incorrectly estimate the effect of built environment attributes at both locations. Empirically, several studies have found that car ownership, commuting mode and commuting distance are all related with the built environment at both ends of commuting (7, 26–30). For instance, in terms of car ownership, Ding and Cao (30) found that the higher employment density and bus stop density at workplace could reduce the likelihood of owning vehicles in the New York metropolitan area, and the residential built environment had a greater impact on the ownership of cars. However, using data from residents in transit-supported suburban neighborhoods in Shanghai, Shen et al. (31) found that there is an insignificant connection between work location and car ownership. Nasri and Zhang (32) found that higher residential and employment densities at residences and workplaces decreased the probability of automobile use and increased the probability of non-motorized travel mode choices. However, Sun and Dan (33) used the Multi-nominal Logistic Regression model and conclude that increasing the population density in residential areas can significantly reduce the probability of private automobile use, while the built environment at workplace has a relatively weak influence on the choice of commuting mode in Shanghai. Dang et al. (28) used a cross-classified multilevel model to estimate the effect of land-use diversity at residences and workplaces on commuting distances and found that land-use diversity at the workplace is more important for reducing commuting distance than at residential areas. Thus, commuting behavior (such as commuting mode and commuting distance) could be affected by both locations. Therefore, we hypothesize that the built environment at both workplace and residential locations should influence CEC, which are closely related to commuting behavior. However, there is still insufficient research about effects of the built environment at both locations on CEC, and it is uncertain regarding the linearity of the relation. Linear models are used widely in the study of the relation between built environment variables and travel behaviors. However, these models cannot solve the multicollinearity between variables, and may cover up the local the non-linear correlation and the threshold effect, which will mislead the planning process (30, 34). Besides, according to previous studies, the built environment variables could have marginal effects on travel behaviors (35, 36). Considering the high density, high mixing degree and the complete public transport system in Chinese cities, the non-linear hypothesis between the built environment and travel behaviors should be adopted in the study of Chinese cities (37). In practice, it is also important for urban planners to explore the most effective impact range of the built environment for low-carbon travel. These research questions will help us understand the mechanism by which built environment variables affect CEC to formulate effective low-carbon urban planning and transportation policies. In addition, socioeconomic factors including income level, education level, and family size, have also been demonstrated to impact travel behaviors (38–41). To fill the gap in the current literature, we use the GBDT model to capture the marginal effects and importance of the built environment variables at residences and workplaces on CEC after controlling for the individual socioeconomic factors.

## Data and methods

### Data

#### Study area

The study area is the urban city of Jinan, which is the capital city of Shandong Province and the central city located within the downstream Yellow River area. As a typical major city in China, Jinan is going through rapid urbanization. For example, the number of household vehicles has increased significantly over the years, from 5.4 per 100 households in 2005 to 58.5 per 100 households in 2020. Similarly, there was an increase in the number of private cars from 0.254 million vehicles in 2005 to 2.962 million vehicles in 2020^1^. The rapid increase in vehicles have negative impacts on traffic and environmental conditions. During the past 5 years, Jinan ranked as the 5th most congested city and the 7th city with heavy air pollution in China^2^^,^^3^. Jinan's overall urban planning calls for low-carbon developments in the new era. Therefore, studying the relationship between the urban built environment and CEC could provide a solid foundation for the development of a low-carbon city.

According to the overall urban plan for Jinan (2021–2030)^4^, this study is conducted primarily in the central area of Jinan, which is located east of the Yufu River, west of the east ring line of the expressway, south of the Yellow River and north of the mountain area. This area encompasses five administrative regions, namely, the Huaiyin District, the Licheng District, the Lixia District, the Shizhong District, and the Tianqiao District. The entire study area is 337 km^2^ and has a population of 2.82 million people which accounts for 77.3% of the urban population in Jinan.

#### Data and variables

The data is obtained from a travel survey from January to July in 2021 for residents over 18 years old. The survey design takes into consideration of error control mechanism and introduces randomness to ensure equal participation opportunities for residents located in each subdistrict. Besides, the number of participants was determined based on the proportion of the population within each district. For 1,200 surveys distributed, the residence address, work address and socioeconomic attributes are sampled at the same time, while participants with no employment information (e.g., students, retirees, and freelancers, are removed from the sample. After applying all filters, our sample contains 920 observations from 64 residential subdistricts and 57 workplace subdistricts.

**Figure 1:**
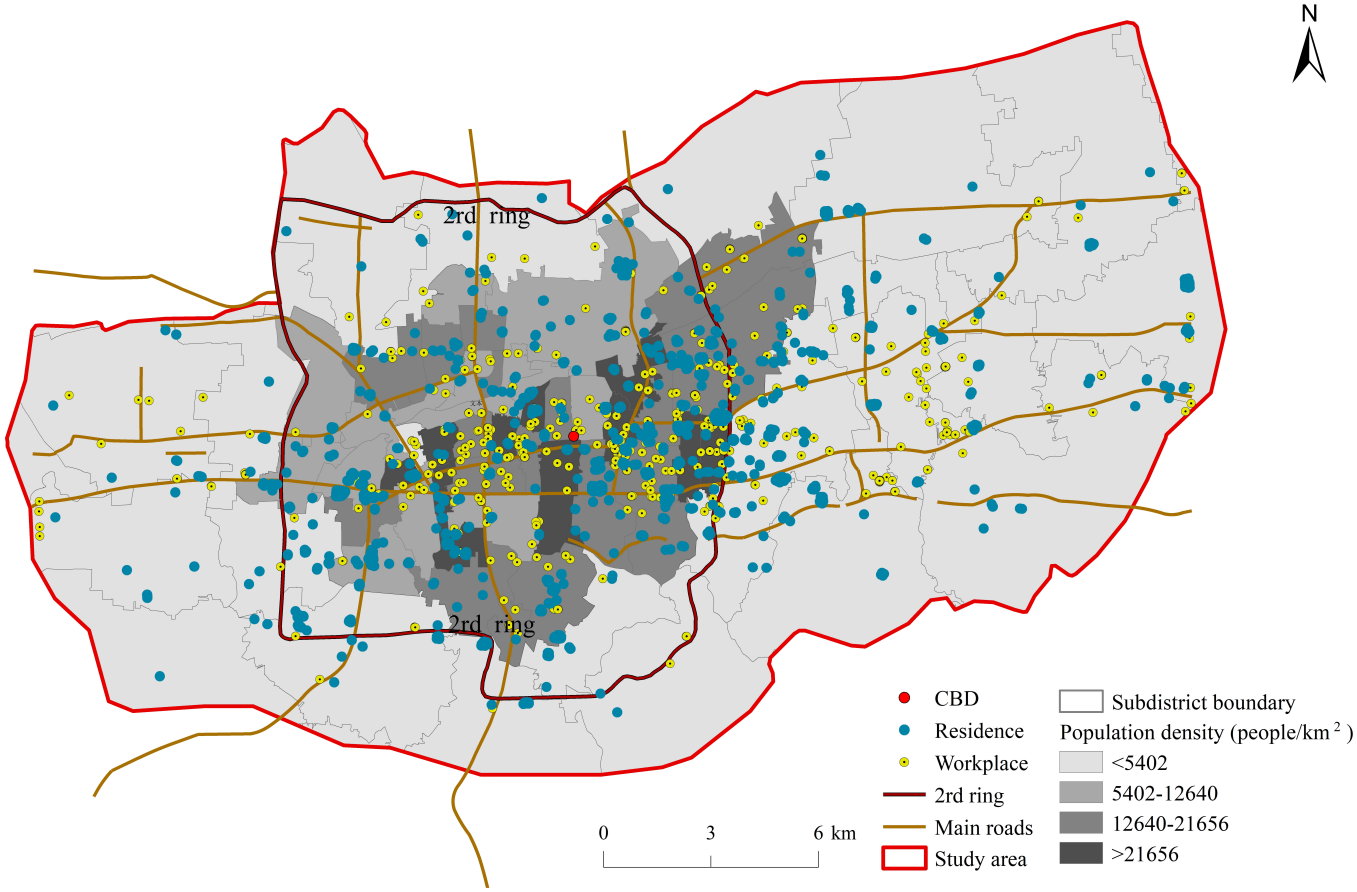
Locations of the residential areas and workplaces of respondents in central Jinan.

To better understand the distribution of geographical locations for the respondents, the location of the residential areas and workplaces are obtained through spatial analysis methods from ArcGIS 10.2 platform. Based on the distribution of the residential and workplace respondents presented in Figure 1, it is obvious that most of the respondents are located within the second ring. The distribution of residences is relatively scattered, and the employment sites are more concentrated in the central business district (CBD). Since the local government moved to the eastern suburb in 2009, the city is in transition from the monocentric to polycentric.

The survey is composed of three parts, i.e., individual travel behaviors, socioeconomic factors and several urban built environment factors. Based on the data, descriptive statistical analyses were performed on the travel behavior characteristics, the residential and workplace-built environments, and the socioeconomic attributes of respondents (Table 1).

**Table 1:** Descriptive statistics for variables.

**Variable**	**Description**	**Mean**	**Min**	**Max**	**Std. Dev**.
**Individual travel behavior variables**			
Commuting mode	0 = Commuting by the other modes (58.83%).	0.412	0	1	0.492
	1 = commuting by car (41.17%)				
Commuting distance (km)	Daily commuting traveled distance (one way)	7.155	0.533	37.237	5.906
**Individual socioeconomic variables**			
Gender	0 = Female (52.28%); 1 = Male (47.72%)	0.478	0	1	0.500
Age (years)	Age	38.270	18	68	8.045
Monthly income (RMB/month)	1 = Below 2000 RMB (19.24%)	2.943	1	6	1.191
	2 = 2000 to 2999 RMB (9.35%)				
	3 = 3000 to 4999 RMB (34.89%)				
	4 = 5000 to 6999 RMB (31.96%)				
	5 = 7000 to 9999 RMB (3.48%)				
	6 = 10000 RMB and above (1.09%)				
Family size (people)	Number of household members	3.263	1	7	0.983
Car ownership	0 = No car (22.04%).	0.92	0	3	0.608
	1 = Owing one car (63.7%)				
	2 = Owing 2 cars (13.59%)				
	3 = Owing 3 or more cars (0.43%)				
**Built environment variables at residential place**			
Distance to city center (km)	Straight line distance from CBD and sub center	7.593	4.913	29.480	3.096
Road density (km/km^2^)	Road length within a 1 km radius of residence	5.029	0.363	11.676	2.295
Distance to bus stop (m)	The nearest distance to bus stops from the residence	221.094	24.329	922.03	151.981
Population density(people /km^2^)	Population/subdistrict area	12136.57	297	32870.5	10078.75
Land-use diversity	Degree of mixing of different land uses in residence^*^	0.713	0.434	0.888	0.102
**Built environment variables at workplace**			
Distance to city center (km)	Straight-line distance from CBD and sub center	6.807	4.913	18.937	2.449
Road density (km/km^2^)	Road length within a 1 km radius of workplace	5691	0.337	12.174	2.362
Distance to bus stop (m)	The nearest distance to the bus stops from the workplace	188.459	19.333	1210.28	119.540
Population density (people /km^2^)	Population/subdistrict area	12303.65	598	32870.5	10728.46
Land-use diversity	Degree of mixing of different land uses in workplace	0.741	0.434	0.888	0.088

920 persons, 64 residential subdistricts, 57 workplace subdistricts. The values in brackets in the description column denote the proportion of each categorical variable.

* $Hland_{i}=\frac{-\sum_{K=1}^{n}p_{K,i}\ln(p_{K,i})}{\ln(K,i)}.$.

Travel behavior characteristics include the daily commute mode and commuting distance of the resident. There are eight commute modes included in this study, which are walking, bicycle, electric bicycle, motorcycle, bus, unit shuttle, private car, and taxi. Approximately 41.17% of the residents choose private cars to travel, and 29.48% of the residents choose walking, bicycles, electric cars or other green travel methods. The remaining residents rely on bus traveling. In our analysis, the commuting mode is a dummy variable, and 1 (0) represents car traveling (other transportation methods). The commute distance is obtained from the Baidu map through identifying the respondent's residence and work location. The results indicate that approximately 48.15% of the residents have a commuting distance of more than 6 km and that the average one-way commute distance is 7.155 km.

The socioeconomic variables of individuals and families considered in this study include age, monthly income, level of education, family size and the number of private cars within the household. Combination of these variables reflects the impacts of family demands, life cycle and travel ability on family travel (19). Our result suggested that 47.93% of the respondents are under the age of 40, and 36.53% have an average monthly income of more than 5000 RMB. Additionally, the average family size of the respondents is 3.263, and 77.96% own car.

With respect to the built environment factors, prior studies suggest that the following five variables should be considered (9, 12, 16, 33) distance from the city center, road density, distance to bus stop, population density, and land-use diversity.

The distance from the city center of Jinan is measured by the average straight distance to the main urban center and sub center, in ArcGIS 10.2 software. The average distance from residential areas is 7.593 km, while the average distance from workplaces is 6.807 km. The population density is calculated by dividing the populations by the subdistrict area and is based on survey data from the sixth census of Jinan. The average residential population density is 12136.57 person/km^2^, and the average population density at the workplace is 12303.65 persons/km^2^. The road density reflects the urban design, which is the sum road length within 1 km radius at residence or workplace, and the average value is 5.029 km/km^2^ and 5.691 km/km^2^, respectively. The distance to bus stop reflects access to bus and is captured by the distance from the location (residence or workplace) to the bus stop. The average distance to bus stop from residential areas and workplaces is 221.094 m and 188.459 m, respectively. The land-use diversity reflects the degree of mixing of different land-use types and is calculated following previous studies (28, 42). The value is between 0 and 1. The larger the value is, the higher the degree of land-use diversity and the greater the balance in the distribution of the land functions. In this paper, the subdistrict is used as the unit of measurement. Based on the land use map of Jinan in 2020, four types of land use which are closely related to residences and work are selected: residential land, public service facilities land, industrial land, and municipal utility land. The average diversity of land use at the residential and workplace levels is 0.713 and 0.741, respectively. Descriptions of the variables in this study are shown in Table 1.

### Methodology

#### Measurement of CEC

To calculate CEC accurately, this study measured CO_2_ emissions based on trip distance methods proposed by current travel research (19, 43–45). Based on transportation modes and the commuting distances obtained through surveys, the commuting CO_2_ emissions could be directly calculated using the following formula:


(1) $$\begin{array}{l} {CE}_{i}=\underset{t}{\sum }\underset{j}{\sum }D_{itj}\times F_{j} \end{array}$$

Where ***CE***_***i***_ is the daily commuting CO_2_ emission of respondent *i*. ***D***_***itj***_ is commuting distance of respondent *i* using the commuting mode *j* for the commute t. And ***F***_***j***_ represents the CO_2_ emission factor of the commuting mode *j*. According to the studies on China's transportation CO_2_ emissions (45, 46), the relevant parameters are presented in Table 2, where CO_2_ emissions are direct emissions.

**Table 2:** Carbon emission factor by transportation modes.

**Transportation mode**	**Transportation** **tool**	**CO_2_ intensity** **(g/person km)**
Car	Private car, taxi,	233
Public transit	Bus,	26
	Unit shuttle	20.3
Personal assistive mobility device	Electric bicycle, light motorcycle	10
Other	Walking, bicycle	0

**Figure 2:**
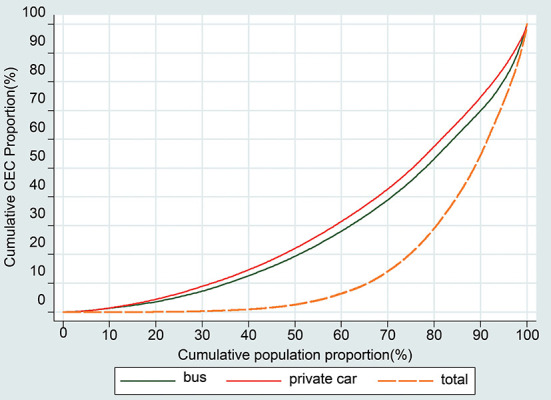
Lorenz curve of the distribution of daily CEC from cars, buses and all respondents in Jinan.

Based on Equation (1), the commuting carbon emissions of each respondent in central city of Jinan could be obtained for a single day. For all respondents, the average value of the commuting CO_2_ emissions is 1641.579 g, and for the respondents commuting by car and by bus, the average value is 3687.849 g and 529.618 g, respectively. Figure 2 shows the Lorenz curve of CO_2_ emissions from private cars, buses, and all respondents in the central city of Jinan. The distribution of the commuting CO_2_ emissions from all respondents is not equal and reflects the 70/20 principle, in which 70% of the CO_2_ emissions are generated by approximately 20% of the residents. For respondents commuting by cars and buses, the distributions approximately fit a 60/30 distribution, indicating that 60% of the CO_2_ emissions are generated by 30% of the residents.

#### The GBDT model

The GBDT model is applied in this study to explore the association between built environment characteristics and CEC. Through building decision trees, GBDT model is popular in dealing with many classification and regression problems. The literature has shown that the GBDT model is a powerful tool to deal with small sample size (9). Compared with the traditional regression models, the GBDT approach cannot produce the statistical inference and the significance level of variables, but it can deal with multicollinearity among variables more effectively, determining the importance of variables, and allowing for accurate predictions (47). The GBDT model can be expressed as follows:


(2) $$F_{M}\left(x\right)=\sum_{m=1}^{M}T(x,\theta_{m})$$

Where ***T***(***x***, **θ**_***m***_) is the decision tree; M is the number of trees, **θ**_***m***_ is the parameters of the decision tree.

In the GBDT model, the loss function of the decision tree is the squared error function, which is denoted by ***L*****(•)**. And the minimum loss function is used to determine the parameters of the next decision tree, where ***T***_***m***−**1**_(***x***_***i***_) is the current decision tree.


(3) $${\hat{\theta }}_{m}=argmin\sum_{i=1}^{N}L[y_{i};T_{m-1}\left(x_{i}\right)+T(x;\theta_{m})]$$

To obtain robust model results, the GBDT model is estimated by R software in this study. Specifically, the sample is divided into five subsets, at each iteration, the model is fitted using four different subsets (80% of the data) and validated by the remaining subset (20% of the data). Overall, a maximum of trees and the shrinkage parameter is set to 1000 and 0.05, respectively. And we chose five-way interaction, and the best results could be obtained after 2,500 boosting iterations. The R squared is 0.221. The relative importance of independent variables and partial dependence plots are derived for further analysis.

## Results

### The relative influence of independent variables

Table 3 presents the relative influence of built environment characteristics and individual socioeconomic variables. The relative influence of a predictor measures its relative empirical improvement in reducing prediction errors. The total relative influence of all predictors adds up to 100%. The built environment is more important in predicting CEC than individual socioeconomic variables. This finding is consistent with other studies that applied the GBDT model to examine built environment effects on driving behavior (8). Specifically, the ten built environment characteristics at residence and workplace collectively contribute to almost 70% of the predictive power, whereas the five individual socioeconomic variables account for about 30%. Besides, the built environment characteristics at workplace contribute to almost 40%, more significant than those at residence, which account for around 30%.

**Table 3:** The relative importance of variables.

**Variables**	**Importance (%)**	**Rank**
Individual socioeconomic variables	(30.502)	
Car ownership	21.299	1
Income	5.285	7
Age	1.825	14
Gender	0.257	15
Family size	1.836	13
Built environment at residence	(29.577)	
Distance to center	10.530	4
Road density	4.253	8
Population density	2.587	11
Distance to bus stop	8.399	6
Land use diversity	3.808	10
Built environment at workplace	(39.915)	
Distance to center	10.908	3
Road density	13.493	2
Population density	9.097	5
Distance to bus stop	4.244	9
Land use diversity	2.173	12

The most important predicting variable is car ownership (21.299%). Owning a car will directly increase the probability of car commuting, resulting in high carbon emissions. Therefore, the impact is relatively large. The second is the road density in the workplace (13.493%). The road density in the workplace reflects the density and connectivity of road facilities and is an important factor affecting CEC. The third is the distance from the workplace to the city center (10.908%), and the fourth is the distance from the place of residence to the city center (10.530%). The distance between living and working places to the city center jointly determines the commuting distance. These two factors reflect the location characteristics, respectively. The importance of location on commuting behavior has been confirmed in many studies (27), with a total of 21.4% contribution. The fifth is the population density of the workplace (9.097%). The concentration of the population in the workplace is an important factor affecting the commuting behavior of residents. The sixth is the distance from the bus stop in the residence (8.399%). The distance from the bus station reflects the service capability of the public transportation facilities in the residence, and depicts the convenience of residents' commuting by bus, which is of high importance. The importance of other variables is similar, and the value is < 5%. In contrast to most studies which showed that the variable of land use diversity has a greater effect on CEC, our results did not discover important relation between land use diversity and CEC. This result could be related to the calculation method at the street scale. At the street scale, the main urban area has a high degree of land use and development, and the average land use diversity at residence and workplace is 0.713 and 0.741, respectively. The homogeneity is strong, resulting in the weak explanatory power when compared with other variables.

### The effects of built environment at the residence and workplace

**Figure 3:**
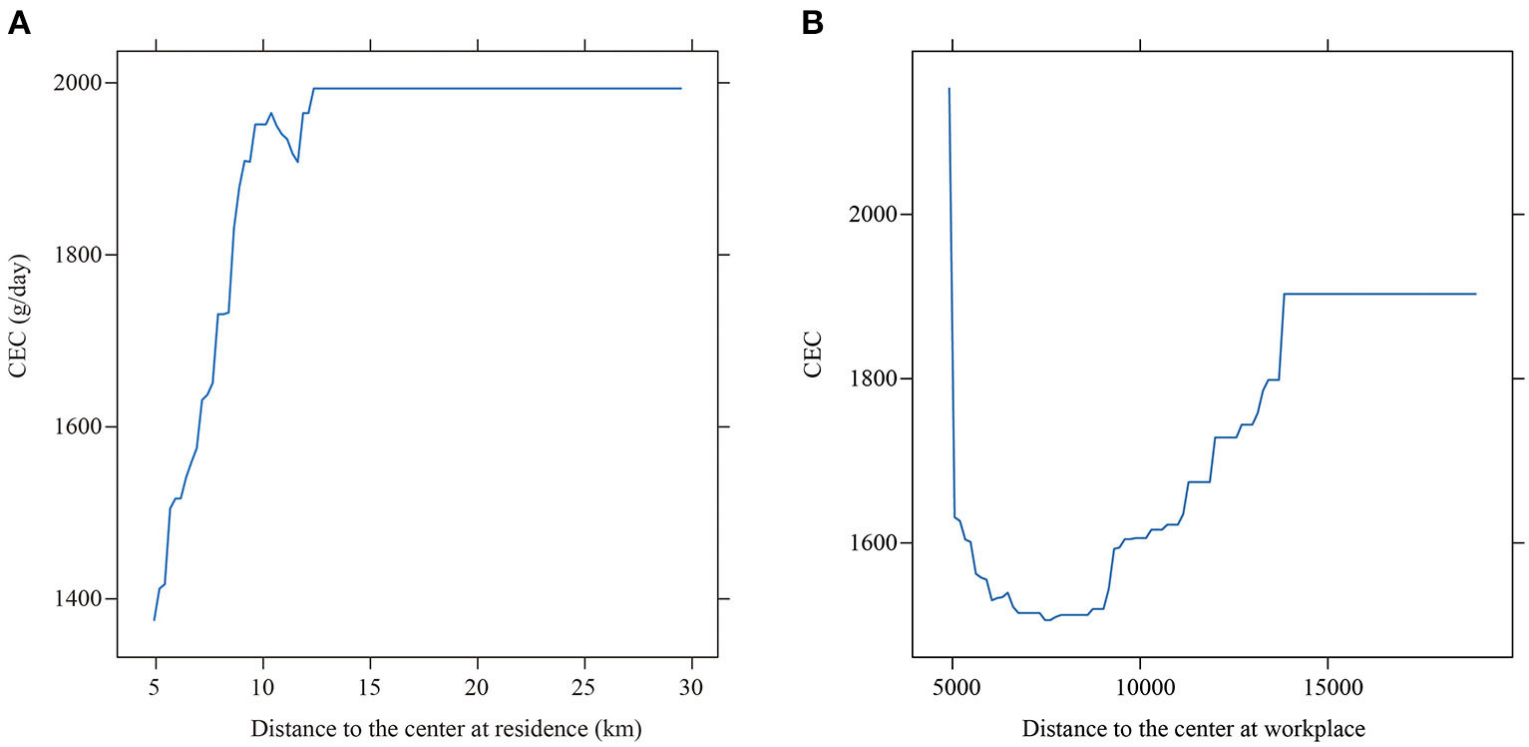
The partial effect of distance to the center on CEC. **(A)** At residence (10.530%). **(B)** At workplace (10.908%).

**Figure 4:**
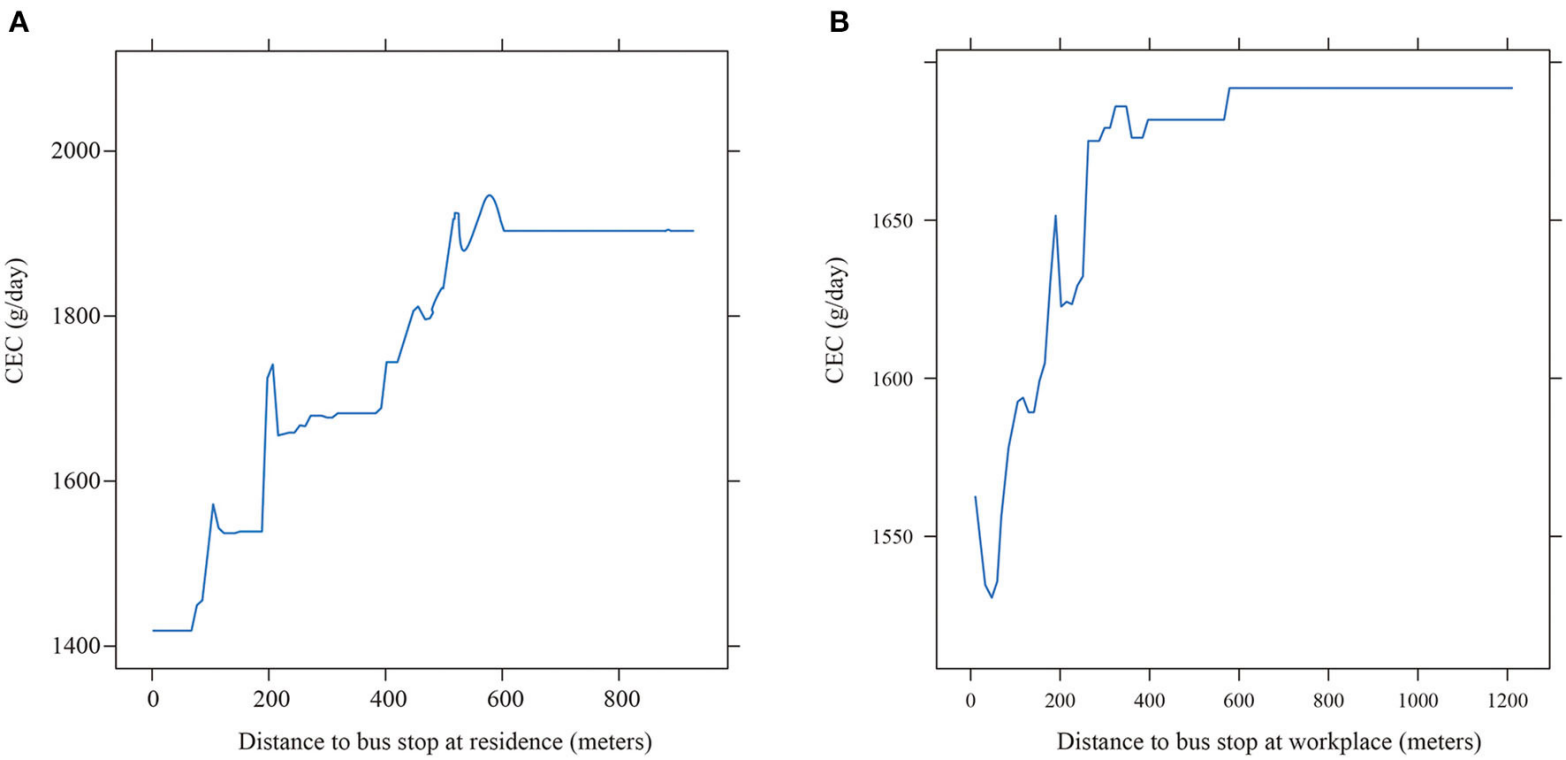
The partial effect of distance to the bus stop on CEC. **(A)** At residence (8.399%). **(B)** At workplace (4.244%).

**Figure 5:**
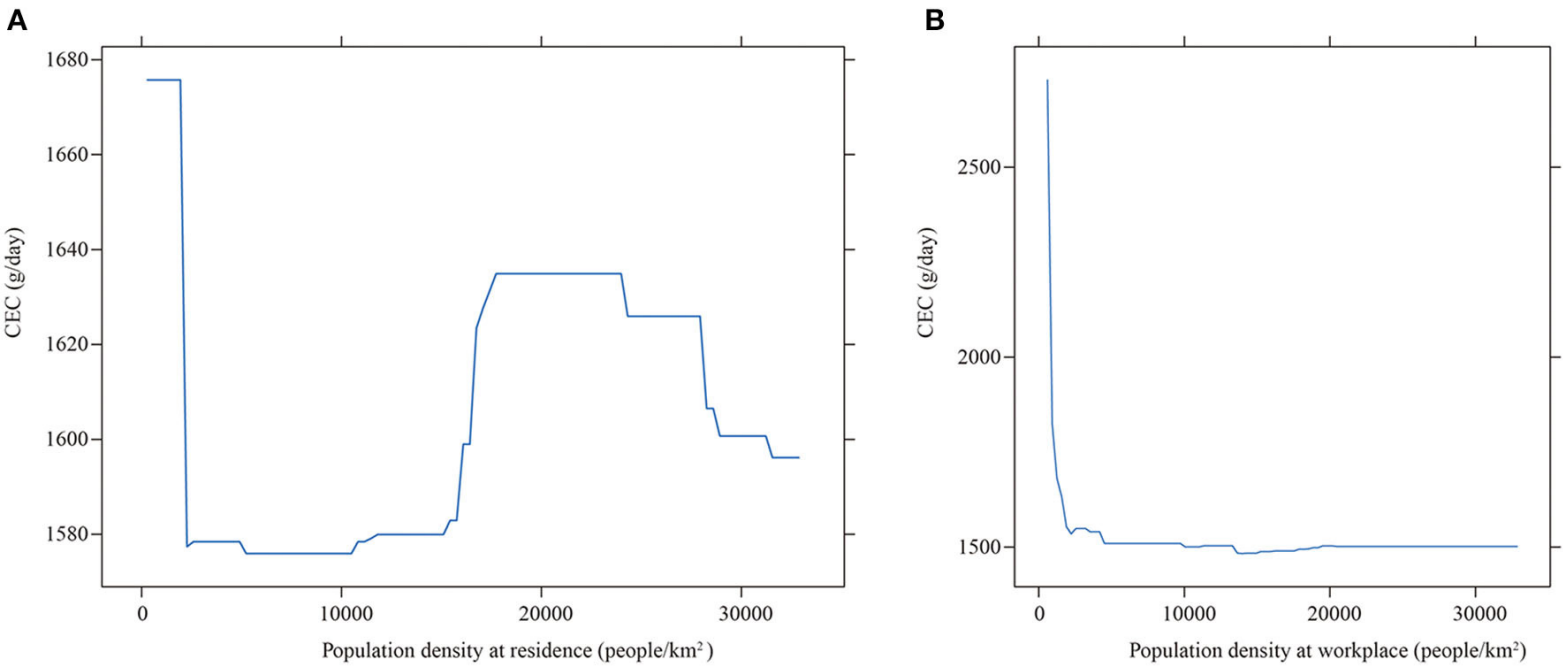
The partial effect of population density on CEC. **(A)** At residence (2.587%). **(B)** At workplace (9.097%).

**Figure 6:**
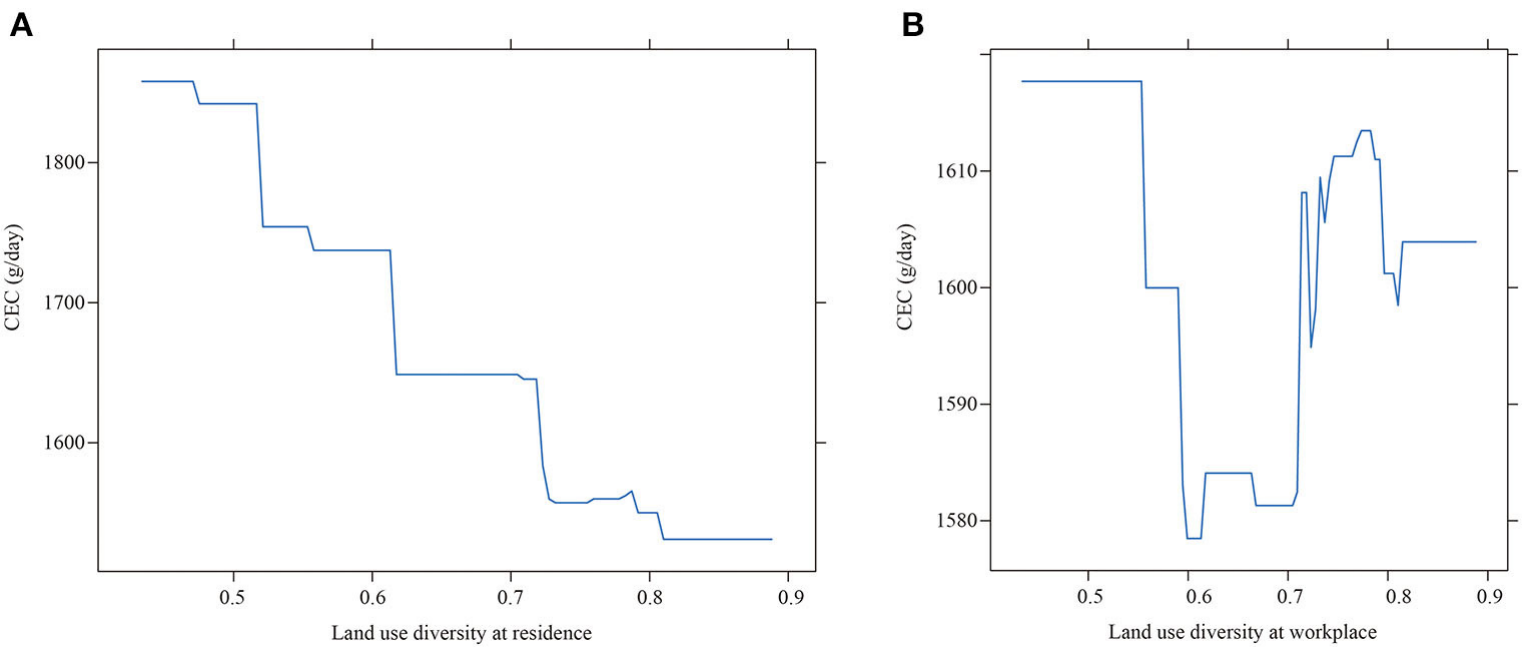
The partial effect of land use diversity on CEC. **(A)** At residence (3.808%). **(B)** At workplace (2.173%).

**Figure 7:**
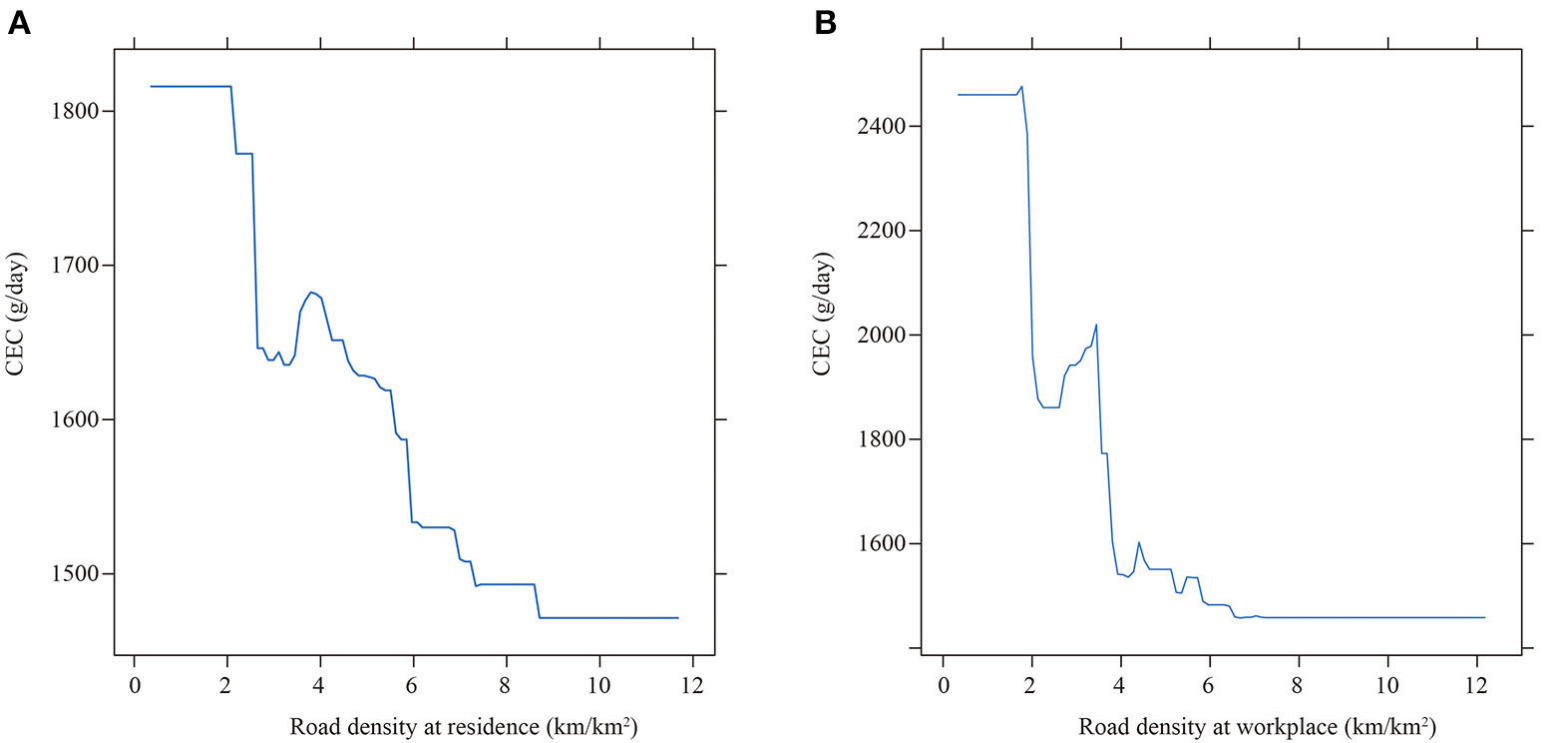
The partial effect of road density on CEC. **(A)** At residence (4.253%). **(B)** At workplace (13.493%).

In the linear model, the coefficient of the independent variable remains unchanged globally, while the independent variable in GBDT does not maintain a stable slope. This may have a non-linear effect on the dependent variable, and there is a threshold effect in the region where the slope changes suddenly. In this way, it can help decision makers find the thresholds of variables and realize the efficient development of urban planning. In this study, we used the GBDT and produced partial dependence plots to illustrate the relationships between built environment variables and CEC (Figures 3–**7**). A partial dependence plot demonstrates the marginal effect of an independent variable on the predicted response while controlling for all other variables in the model. The vertical axis is CEC, the horizontal axis represents the respective variable. The fitted curve is smoothed to better show the changes. The overall trend of all independent variables is consistent with our expectations.

Figures 3A,B illustrates the impact of distance to center, with the relative importance presented along with the label for the horizontal axis. Distance to city center has positive threshold effects on CEC. When the distance is < 12 km, its slope is relatively steep. Once it exceeds 12 km, its slope becomes smooth. When people live close to the city center, the accessibility to facilities is high and people have more travel mode choices instead of driving, and the CEC would be lower. However, for people live far from the city center, they tend to commute by car due to the lower accessibility and inconvenient transit service. Therefore, CEC would be higher. However, once the distance is beyond 12 km, the CEC remains relatively unchanged.

By contrast, distance from workplace to center also takes on non-linear effects on CEC, which decreases before increases. The results show that individuals who work closer to the center within 5 km tend to emit more CEC, since they often reside far away from the center due to the tradeoff between the commuting cost and housing price (32). And according to the survey, the negative correlation are also found between the workplace location and residence location. Since most cities like Jinan in China are still monocentric, there are gradient characteristics such as population density, employment density and housing prices (5). In our sample, 21.3% of those who work within 5 km from the urban center have to commute 10 km or more distance. Thus, people tend to work in close proximity to the center, while residences are far from the center. As the distance increasing, the job-home separation could be improved, and many people will lower the CEC. However, once the distance exceeds 5 km, the CEC shows an increasing trend. The longer distance from the workplace to the city center, the higher dependence on the car when commuting, resulting in an increasing CEC. Similar to the variable of distance to the center at residence, once the distance exceeds 13 km, CEC remains unchanged. The results show that the location of residence and workplace jointly affect CEC, and it is important to form the polycentric spatial structure in central city of Jinan in the future.

Figure 4A illustrates the relation between CEC and distance to bus stop at residence. Overall, the higher accessible transit service has a negative relation with CEC. When the nearest distance to the public transit is <500 meters of a residence, the increase of the distance to bus stop leads to a higher CEC. This pattern is consistent with Gallivan et al. (48). Beyond this range, the change of CEC is trivial as the distance increases. Figure 3B shows the impact of the distance to bus stop at workplaces, and is similar to that at residence which means that better access to bus stop at workplace could lower CEC. This result is consistent with previous research (45). However, once the distance exceeds 500 meters, the change of CEC is insignificant. Therefore, the distance to bus stop at residences and workplaces plays an important role in CEC. The access to public transit at the residences has a significant impact on CEC. This is due to the uneven spatial distribution of bus facilities. Dense bus routes are highly concentrated in the employment center instead of the residences (9).

Figures 5A,B shows the influences of population density. It shows the threshold effect of population density at residence on CEC. Overall, the effect of population density at residence reaches a high level at about 22,000 people/km^2^ before slowly decreases. This could be explained by the concentration of human activities because of high density (36). For most cities in China, locations with low population density often coincide with an inconvenient public transportation and less employment opportunities, which means that residences in these areas are more likely to commute by car. As the population density increasing, the government will improve the supply of public facilities and infrastructures, indicating that there would be a higher usage of the public transport system and less CEC. However, with the further increase of population density, public infrastructure will eventually fail to meet public needs, and car commuting would increase. Once the population density reaches above 22,000 people/km^2^, the issue of traffic congestion would emerge. Preference for the public transport increases, which results in less CEC. In general, there exists a U-shaped relationship between the population density at residence and the CEC. Figure 5B suggests that the increase in population density at the workplace also lowers the CEC. The reason is that urban centers are important areas of employment, and there are normally a complete public transportation system and high population density in these areas. When the population density of workplace increases, traffic jams and high parking charges will occur. Besides, commuting by car is more energy and time consuming. As a result, an increase in the population density at the workplace will reduce the likelihood of car commuting, as well as the corresponding CEC.

As a diversity indicator, land use entropy index at residence is negatively correlated with CEC in Figure 6A. This negative relationship is consistent with the previous literature (49, 50). However, the land-use diversity at the workplace also has a non-linear effect on CEC, and 0. 55, 0.7 and 0.75 are the important turning points. When land use diversity is relatively homogeneous (entropy < 0.55), it has a trivial influence on CEC. However, when there are several types of land-uses (0.55≤entropy≤0.7), CEC decreases. Moreover, land use diversity has increasing effect once all types of land use are relatively evenly distributed (entropy > 0.7). On the other hand, if the land use diversity is higher than 0.75, it will lower the CEC. Overall, land use diversity is effective on CEC when it reaches a certain level, but it has a diminishing return once it reaches a different threshold. The reason is that to give full play to the agglomeration benefits of land, the land use types are often dominated by industrial land and commercial land, and residential land is often distributed in peripheral areas with few job opportunities. This results in a higher degree of home-work separation and more CEC.

Road density could reflect the street connectivity. The literature suggests that it is negatively related to driving distance and positively related to transit use (16). Thus, road density could lead to the decrease of CEC. As shown in Figure 7, the road density at residence within 1 km buffer of residence shows a negative correlation with CEC, similar to that of workplace. It hugely lowers CEC when the road density at residence is higher than 2 km/km^2^, but the decreasing effect is trivial once the road density is higher than 8 km/km^2^.

### The comparison with log-linear regression

A traditional log-linear regression model is constructed to compare with the GBDT model, and natural logarithmic transformation is performed on variables with positive or negative skew distribution, which can effectively improve the fitness of the linear model. To achieve desired results, the natural logarithm standardization was performed on all continuous variables. Analyzing the results of OLS model estimation in Table 4, it can be found that the *p*-value of the F test is < 0.001, indicating that the results are reliable. The standardized coefficients of the variables show the relative agreement between the traditional model and the GBDT model on the overall expected effect, but the significance and the relative importance of the variables of the GBDT model are quite different. Specifically, we include the built environment variables of residence and workplace in the regression model, respectively, and results show that the significant variable either at residence or at workplace could be found. However, after including all variables in the regression model, the variable at workplace (distance to center from workplace) becomes insignificant. These results confirm that the linear regression could cover up the local impact of variables. Besides, the *R*^2^ of the log-linear regression is 0.137, which is lower than that of GBDT (0.223), indicating that the GBDT method is more suitable for explaining the impact of built environment variables on CEC. The difference is due to the biased estimation caused by the pre-existing linearity assumption, reflecting the advantages of non-linear models.

**Table 4:** The results of log-linear regression.

**Variables**	**(1)**	**(2)**	**(3)**
**Individual socioeconomic variables**			
Age	−0.024^**^	−0.026^**^	−0.024^*^
	(0.012)	(0.012)	(0.012)
Gender	0.250	0.251	0.238
	(0.191)	(0.194)	(0.193)
Income	0.242^***^	0.217^**^	0.240^***^
	(0.087)	(0.089)	(0.088)
Car ownership	1.447^***^	1.443^***^	1.447^***^
	(0.165)	(0.165)	(0.165)
Family size	0.230^**^	0.197^**^	0.227^**^
	(0.094)	(0.095)	(0.095)
**Built environment at residence**			
Distance to center	1.172^***^		1.110^***^
	(0.360)		(0.402)
Road density	0.065		0.035
	(0.252)		(0.258)
Population density	−0.013		0.005
	(0.091)		(0.094)
Distance to bus stop	0.290^**^		0.288^**^
	(0.127)		(0.128)
Land use diversity	−0.113		−0.114
	(1.677)		(1.720)
**Built environment at workplace**			
Distance to center		0.742^**^	0.087
		(0.373)	(0.416)
Road density		0.118	0.035
		(0.247)	(0.250)
Population density		−0.088	−0.062
		(0.075)	(0.077)
Distance to bus stop		0.005	0.015
		(0.139)	(0.138)
Land use diversity		−0.288	−0.335
		(1.836)	(1.859)
Constant	−7.464	−1.773	−7.076
	(4.869)	(4.877)	(6.070)
Observations	920	920	920
R^2^	0.137	0.118	0.137
F	14.29^***^	12.05^***^	9.59^***^

*** ,

** and

* indicate that the p-value is significant at the 1, 5 and 10%, respectively.

Figures in brackets denote standard errors.

## Discussion and limitations

### Accuracy of the GBDT model

The GBDT model is adopted in this study to examine the non-linear relationship between built environment characteristics and CEC at residence and workplace. In contrast to the parametric specification of non-linear relationships, this model considers the non-linear relationships between built environment variables and CEC. It also assesses the relative importance of different built environment characteristics in reducing CEC and the collective contribution of built environment variables relative to individual socioeconomic characteristics. Since the patterns of non-linear relationships vary among built environment variables, this makes parametric specification of non-linear relationships inefficient and inaccurate. Apart from that, it is suggested that applying the threshold of built environment variables during the urban planning process would help achieve the goal of a low carbon city (51).

### The effects of built environment

Consistent with prior research, our results confirmed that the built environment has stronger impacts on CEC compared with the individual socioeconomic variables. Compared with built environment variables at residence, those at workplace have affect CEC significantly, indicating that the built environment characteristics of work center can be used to meet the low carbon development goal in the future. For example, according to the current urban spatial structure of Jinan, employment land is concentrated in the urban center and residential land is spread outward. This structure offers individuals employed in the central urban area a wider range of locations to choose their residences.

The five built environment variables together, i.e., the road density at workplace, the distance to the center at workplace, the distance to the center at residence, the population density at workplace and the distance to bus top, contribute to more than 5% of CEC. Among all variables, the distance to the center at residence and workplace, representing the regional location, played the most important role in reducing CEC. The regional location largely determines land use characteristics and transportation infrastructure surrounding the region (20). On the other hand, land use diversity tends to be less influential. This is different from research findings in Western countries, which attached more importance to the effect of high land use diversity and high population density in reducing CEC (9). While in China, majority of big cities are characterized by dense population, high land use diversity and complete public transport networks. Therefore, the regional location will have a stronger impact on CEC than other variables in China.

Specifically, the road density at workplace has the strongest impact on CEC. It lowers CEC significantly when the road density is higher than 2 km/km^2^, once the road density is higher than 8 km/km^2^ the decreasing trend becomes trivial. This indicates that road density is not negatively related to CEC under all circumstances.

The distance to the center at residence and workplace indicates the local location, which jointly contribute to 20% of the total CEC. In general, due to the dislocation of workplace and residence, the distance from the city center is positively related to the level of CEC. However, according to the threshold effect, when the distance to center reaches 12 km, the CEC remains high, indicating that the government could formulate the industry layout within such distance.

The increase in population density at workplace also has a negative impact on CEC. This implies that urban planners could increase residential land supply in the employment center accordingly to alleviate home-work separation, meanwhile raise awareness of compact development. This could increase the mixed functionality of urban land and create more employment opportunities nearby, which could effectively reduce the reliance on commuting by cars, lowering the CEC accordingly.

The distance to the bus stop at residence which represents the public transit accessibility also plays a vital role in determining CEC. Many studies have also confirmed that the higher the accessibility of public transport, the more likely residents are to choose non-motorized commuting. Individuals who live in longer distance from the bus stop will emit more CEC. As the distance from residence to the bus stop increases, the demand for taking the bus decreases and many people will prefer to commute by car. Consistent with previous studies (21, 25), the more commute they take, the higher CO_2_ emissions there will be (44). Therefore, it is necessary to solve the last mile problem during commuting to effectively limit the demand for car commuting, and reduce CO_2_ emissions. These results can help planning practitioners effectively prioritize objects for urban built environment intervention.

The findings in the study have direct policy implications for Chinese cities like Jinan, which are in the process of rapid motorization.

First, this study shows that if planners focus on future population and employment growth in central urban areas (or up to 12 km from centers), the amount of increase in CEC associated with population and employment growth will be minimized. Multicenter development is actively advocated to provide more job opportunities, especially in outer areas of the central city, to shorten the commuting distance as well as reduce the related CO_2_ emissions. Second, in terms of land use planning, urban planners should pay more attention to the built environment variables at workplace. This would help to improve the road network quality at employment centers. Additionally, due to the high level of land use diversity in employment centers, continuously increasing the land use diversity will not lead to a reduction in CEC. However, it should appropriately increase the supply of residential land in the employment center to alleviate the home-work separation. Third, it is important to improve the transportation access at both residence and workplace to decrease CEC, and solve the problem of last mile during commuting, enhancing the attractiveness of public transportation. This is especially the case for those who prefer to commute by car, and reduce potential CEC. In addition, multimodal transportation systems and more plausible land-use patterns should be established to support sustainable urban development, such as acceleration of the construction of multiple urban public transport modes (e.g., subway and BRT), and managing the personal commuting activities through big data and information technology.

### Limitations

This study has the following limitations. First, the computation of CEC was based on existing emission factors, and we did not consider vehicle or transit occupancy, which could affect the true CEC from commuting. Second, the population density and land-use diversity are measured at subdistrict level. However, the effect of the built environment variables is measured at different spatial scale (such as traffic zone, community, and 1 km or 500 m radius of a residence). In addition, policymakers are more concerned about which dimensions of the built environment can lower CEC. Therefore, the comparative study of the effects of variables at different scales requires further study in the future; Third, in terms of the power of the CO_2_ emission from commuting model, it could be improved by incorporating additional built environment variables, such as road network design, employment density, and parking availability, into the models for a more in-depth analysis. Lastly, since the data are cross-sectional, the influence found in this study is more of an association than causality, similar to most studies in the literature. In general, this study helps to understand the impact of built environment variables at both residences and workplaces on the CEC to large cities of China.

## Conclusions

Based on the assumption of non-linear relationship between built environment and travel behavior, this study applies the GBDT model in analyzing the impact of the built environment at both residences and workplaces on CEC using the daily travel survey data of residents in the central city of Jinan. After controlling the socioeconomic factors, we examine the non-linear threshold effect of each variable on CEC, which enriches the existing theoretical and empirical research. Our findings suggest that:

(1) Built environment variables collectively are more important in predicting CEC than individual socioeconomic variables, which is consistent with most studies using parametric models. The following five variables have the highest predicting power among built environment characteristics: road density at workplace (13.493%), distance to the center at workplace and residence (10.908 and 10.530%, respectively), population density at workplace (9.097%), and distance to bus stop from the residence (8.399%). The distance to the center at residence and workplace, representing the local location, jointly contribute to 20% of CEC. In terms of the socioeconomic variables, car ownership has the highest predictive power (21.299%). On the other hand, land use diversity tends to be less influential either at residence or at workplace.

(2) The built environment at workplace contributes to 40% of the total CEC, which is higher than the build environment at residence. It is necessary to perform a planned intervention of the built environment elements at both residence and workplace. Majority of built environment variables at residence had similar impacts on CEC as those of workplace except population density and land use diversity, the impact of which on CEC varies significantly between the residence and workplace. Contrary to the impact of land-use diversity at residence, there are threshold effects for the land-use diversity at workplace affecting CEC. For the population density, the threshold effect only exists for residence.

(3) The non-linear and threshold effects of the urban built environment and CEC are determined in the Jinan city, and the threshold value of built environment variables could be obtained using the GBDT methods, which could guide the urban layout in future during the low carbon city construction.

## Data availability statement

The raw data supporting the conclusions of this article will be made available by the authors, without undue reservation.

## Author contributions

Conceptualization: JY. Methodology and formal analysis: QL and PZ. Data curation: QL, PZ, and YZ. Writing—original draft preparation and writing view and editing: QL and ZZ. Visualization and software: PZ. Project administration, validation, and funding acquisition: QL. All authors have read and agreed to the published version of the manuscript.

## Funding

This work was funded by the National Natural Science Foundation of China for Grant Support (No. 41401163); Humanities and social science projects, Education Ministry of China (No. 19YJCZH107); and Natural Science Foundation of Shandong Province (No. ZR2020MD011).

## Conflict of interest

The authors declare that the research was conducted in the absence of any commercial or financial relationships that could be construed as a potential conflict of interest.

## Publisher's note

All claims expressed in this article are solely those of the authors and do not necessarily represent those of their affiliated organizations, or those of the publisher, the editors and the reviewers. Any product that may be evaluated in this article, or claim that may be made by its manufacturer, is not guaranteed or endorsed by the publisher.

## Footnotes

^1^Source: Bureau of Statistics of Jinan. (2002–2018) Jinan Statistical Year book. Beijing: China Statistic Press, China.

^2^Source: Amap.com. China Major Cities Transportation Analysis Report 2018 Q3. 2018. Available online: https://trp.autonavi.com/traffic/ (accessed on March 15, 2019).

^3^Source: Ministry of Environment Protection (MEP), 2018. Rank of national key monitoring cities air quality year. Environ. Educ. Z1, 16.

^4^Source: Jinan Municipal Planning Bureau. Jinan Urban Development Strategic Plan (2018-2050) [2019–04–02]. Available online: http://nrp.jinan.gov.cn/art/2019/4/2/art_43830_3510946.html (accessed on April 2, 2019).
